# Role of Intraoperative Nerve Monitoring in Preventing Peripheral Nerve Injury During Total Hip Arthroplasty in High-Risk Patients

**DOI:** 10.7759/cureus.80233

**Published:** 2025-03-07

**Authors:** Teiji Harada, Hiroshi Iwasaki, Takaya Taniguchi, Wataru Taniguchi, Daisuke Nishiyama, Daisuke Fukui, Manabu Yamanaka, Hiroshi Yamada

**Affiliations:** 1 Department of Orthopaedic Surgery, Wakayama Medical University, Wakayama, JPN

**Keywords:** developmental dysplasia of the hip (ddh), free-run electromyography, iatrogenic nerve injury, intraoperative nerve monitoring (ionm), motor evoked potential, total hip arthroplasty (tha)

## Abstract

Background

Intraoperative nerve monitoring (IONM) plays a crucial role in preventing peripheral nerve injury during total hip arthroplasty (THA). However, its efficacy in THA remains controversial. This study aimed to evaluate the efficacy of IONM in preventing peripheral nerve injury during THA.

Methods

This study retrospectively included 79 hips in 72 patients (primary THA: 43 hips; revision THA: 36 hips) with risk factors for intraoperative nerve injury who underwent motor evoked potential (MEP) monitoring combined with THA between July 2011 and March 2019. A concerning MEP change was defined as a decrease to <30%. The frequency and cause of the MEP change and the presence of postoperative neurological symptoms were investigated.

Results

Motor evoked potential changes were detected in five of the 79 hips (6.3%). In one of these cases, the potential amplitude did not recover, and transient postoperative muscle weakness was observed due to the delayed detection of MEP changes. In the remaining cases, the potential change was quickly detected by IONM, including the regular-interval monitoring method, without any subsequent postoperative muscle weakness.

Conclusions

Intraoperative nerve monitoring effectively prevented peripheral nerve injury during THA, particularly in patients with developmental dysplasia of the hip (DDH) or a history of hip surgery. To prevent irreversible postoperative neuropathy, regular interval nerve monitoring, and free-run electromyography should be considered routine techniques in THA for patients with DDH or a history of hip surgery.

## Introduction

Peripheral nerve injury during total hip arthroplasty (THA) is an uncommon but devastating complication. The potential causes of nerve injury include direct trauma, excessive tension due to leg lengthening, ischemia, and compression by hematoma [1-4]. Several risk factors have been suggested, including female sex, developmental dysplasia of the hip (DDH), revision surgery, posttraumatic arthritis, cementless femoral fixation, anatomical variation, and lumbar spine disease [1-9]. The incidence of peripheral nerve injury during THA is reported to be approximately 1% [2-7]; however, in cases with risk factors, the incidence increases from 1.4% to 7.6% in revision cases [3, 4, 7, 10] and 5.7% to 10.0% in DDH cases [11-13]. The majority of peripheral nerve injuries during THA, whether complete or incomplete, do not recover to their preoperative nerve status [4, 6]. The persistence of paralysis forces these patients to undergo prolonged treatment, and they may require canes or braces. These patients have limited postoperative activity and less satisfactory clinical outcomes. Therefore, it is crucial to prevent peripheral nerve injury during THA.

Intraoperative nerve monitoring (IONM) is a useful tool that can provide instant and accurate alerts for relevant nerves during surgery, which facilitates the modulation of actions to prevent iatrogenic nerve injuries [14]. Recent studies have demonstrated the usefulness of IONM in hip surgery [15-18]. In our institution, transcranial motor evoked potential (MEP) monitoring is routinely performed during THA for patients with risk factors for peripheral nerve injury. This study retrospectively evaluated the effectiveness of IONM in preventing peripheral nerve injury during THA.

## Materials and methods

This retrospective study was conducted in accordance with the principles of the Declaration of Helsinki. The study design was approved by the institutional review board of Wakayama Medical University, Wakayama, Japan, and informed consent was obtained from the participants prior to their inclusion in this study. Between July 2011 and March 2019, 407 patients underwent primary THA, and 70 patients underwent revision THA at our institution. Among them, MEP monitoring was performed during THA in patients with risk factors for intraoperative nerve injury, including DDH, posttraumatic arthritis, revision surgery, a history of hip surgery, and planned leg lengthening >2 cm.

The MEP was recorded by conducting transcranial stimulation following the method of Matsuda and Shimazu [19]. A pair of corkscrew electrodes (001-220; Agram, NJ, USA) were symmetrically placed on the scalp 5 cm outside and 2 cm forward from Cz (International 10-20 System of Electrode Placement), overlying the motor cortex area of the skull. The stimulation was applied using a constant-current stimulator (MS-120B, Nihon Kohden, Tokyo, Japan) with a train of ﬁve biphasic stimuli, each lasting 0.5 ms (two phases of 0.25 ms in each stimulus) and an inter-pulse interval of 2 ms. The MEPs were obtained from a pair of needle electrodes (NE-220B; Technomed Europe, Amerikalaan, The Netherlands) placed in the quadriceps femoris (femoral nerve), tibialis anterior (peroneal nerve), and abductor hallucis (tibial nerve) muscles.

The operations, under general anesthesia, were performed by experienced hip surgeons. The anesthesia protocol involved total intravenous anesthesia using propofol and remifentanil. A muscle relaxant was administered only at anesthesia induction to facilitate tracheal intubation, and an antagonist was administered before the monitoring procedure to eliminate the effects of the muscle relaxant on nerve conduction. Inhalational anesthetics, such as isoflurane and nitrous oxide, were not used because they attenuate the amplitudes of MEP [20]. The MEP monitoring was performed throughout the surgical procedure. The alarm point of the potential amplitude decrease was defined as 30% (a 70% decrease) based on the standard values in spinal surgery [21]. When the alerts occurred, the surgeon investigated and addressed the cause of MEP change.

The frequency and cause of MEP change and the presence of postoperative neurological symptoms were investigated. Neurological symptoms were examined within 24 hours after the surgery. If neurological symptoms were observed, follow-up assessments were conducted to monitor nerve recovery.

## Results

A total of 79 hips from 72 patients (13 men and 59 women; primary THA: 43 hips; revision THA: 36 hips) were included in this study. The mean age of the study population at the time of surgery was 67.0 years (range: 41-84 years). The preoperative diagnosis for primary THA included DDH (22 hips, five of which had a history of hip surgery), residual Perthes-like deformity (eight hips, one with a history of hip surgery), osteonecrosis (five hips), rheumatoid arthritis (three hips), rapidly destructive coxopathy (three hips), posttraumatic arthritis (one hip), and postinfectious arthritis (one hip). The revision surgeries were performed due to infection (10 hips) and aseptic loosening (26 hips) (Table 1).

**Table 1:** Characteristics of patients included in the study SD: standard deviation

Characteristics	
Sex, n (%)	
Male	13 (18.1%)
Female	59 (81.9%)
Age (years; mean ± SD)	67.0 ± 11.2
Diagnosis (number of hips)	
Developmental dysplasia of the hip	22
Residual Perthes-like deformity	8
Osteonecrosis	5
Rheumatoid arthritis	3
Rapidly destructive coxopathy	3
Posttraumatic arthritis	1
Postinfectious arthritis	1
Revision due to infection	10
Revision due to aseptic loosening	26

Alerts indicating a potential amplitude decrease were detected in five of the 79 hips (6.3%) (Table 2). Four cases involved primary THA for DDH (two of which had a history of hip surgery), and one involved revision THA. The causes of the alerts were direct trauma by a retractor (two cases), leg lengthening (two cases), and low blood pressure due to anesthesia (one case). There were no cases of permanent postoperative neuropathy.

**Table 2:** Characteristics of the patients with motor evoked potential alerts DDH: developmental dysplasia of the hip; THA: total hip arthroplasty

Age	Sex	Preoperative diagnosis	Surgery	Causes	Interventions	Outcome	Postoperative symptoms
77	F	Aseptic loosening	Revision	Compression by retractor	Repositioning of retractor	Not recovered	Muscle weakness
56	F	DDH	THA	Compression by retractor	Repositioning of retractor	Recovered	None
50	F	DDH with previous hip surgery	THA	Leg lengthening	Shorter prosthesis	Recovered	No muscle weakness (Sensory disturbance)
55	F	DDH with previous hip surgery	THA	Leg lengthening	Shorter prosthesis	Recovered	None
60	F	DDH	THA	Low blood pressure	Vasopressor administration	Recovered	None

**Figure 1:**
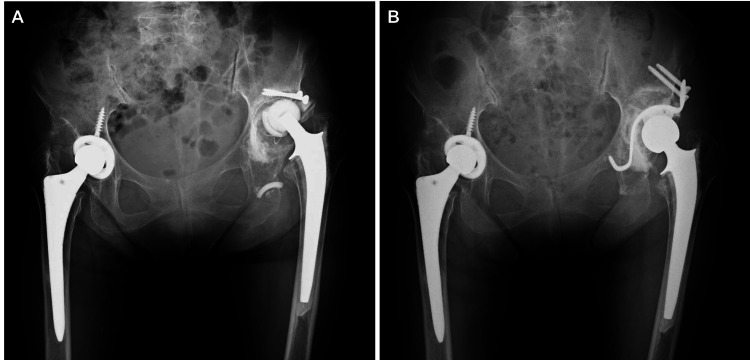
Radiographic images (A) Preoperative and (B) postoperative radiographic images with an anteroposterior view of the bilateral hip joint. For the loosening of the acetabular component with a bone defect, revision total hip arthroplasty was performed with acetabular reconstruction.

**Figure 2:**
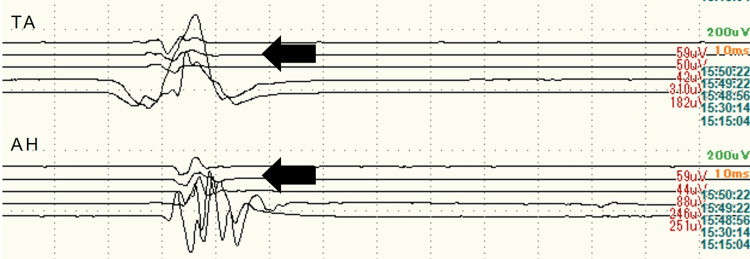
Waveform of motor evoked potential (MEP) after acetabular reconstruction Decreased potential amplitude is observed in TA and AH on the affected side (black arrow). TA: tibialis anterior; AH: abductor hallucis

**Figure 3:**
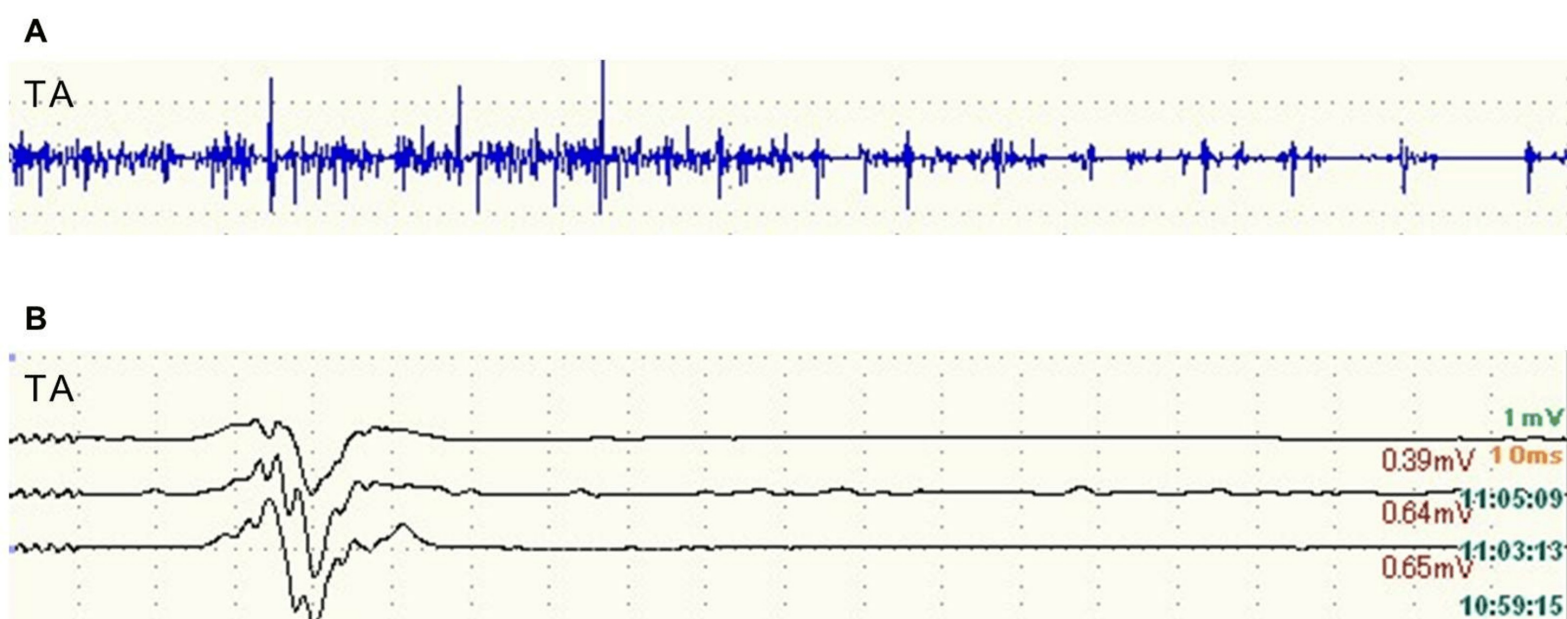
Waveforms of free-run electromyography (fEMG) (A) and motor evoked potential (MEP) (B). The fEMG of TA shows bursts of sharp waves, and a decrease in MEP amplitude is observed in TA (black arrow). TA: tibialis anterior

In one case where an MEP alert was caused by a retractor, the potential amplitude did not recover even after repositioning the retractor, and the amplitude remained below 30% at the end of the surgery. This patient was a 77-year-old woman who underwent revision THA for aseptic loosening of the acetabular component (Figures 1A, 1B). After acetabular reconstruction, the potential amplitude decreased at the tibialis anterior and abductor hallucis on the affected side (Figure 2). The compression of the sciatic nerve by the acetabular retractor at the posterior wall of the acetabulum was considered the cause of the potential amplitude decrease. Despite the removal of the posterior retractor, the amplitude of MEP did not revert to normal. In this case, it took time to detect the potential change caused by retractor-induced compression because MEP monitoring was performed only at specific points. As a result, the potential amplitude did not recover even after the retractor was removed. Postoperatively, muscle weakness of the tibialis anterior and abductor hallucis on the affected side was observed, which gradually improved from the second postoperative day and recovered completely four weeks after the surgery. Based on the learning experience from this case, MEP monitoring was performed at regular intervals throughout surgery. In addition, free-run electromyography (fEMG) was incorporated to promptly detect changes in nerve status. In another case, these continuous nerve monitoring techniques allowed for the immediate recognition of nerve compression caused by the retractor (Figures 3A, 3B). The potential amplitude recovered after the retractor was repositioned, and no postoperative neuropathy occurred.

In the cases of nerve injury caused by leg lengthening, the potential amplitude decreased to less than 30% when the planned amount of leg lengthening was performed. Therefore, the surgeons reduced the amount of leg lengthening, and the potential amplitude recovered to more than 30% in both cases. Owing to the procedure, although a temporary sensory disturbance was observed in one case, no postoperative muscle weakness was observed in both cases.

In the case of nerve injury due to anesthesia-induced hypotension, the anesthesiologist administered a vasopressor, and the potential amplitude reverted to normal without any changes to the surgical procedure.

## Discussion

Nerve monitoring techniques have been developed primarily in spinal surgery and include multimodal techniques, such as somatosensory-evoked potential (SEP), electromyography, MEP, and others. In THA, the efficacy of IONM for preventing postoperative neuropathy remains controversial. Several studies have reported similar incidences of postoperative neuropathy between patients who underwent IONM and those who did not [22,23]. However, many investigators have recommended nerve monitoring as an effective method for preventing iatrogenic nerve injury [15-18]. In our institution, IONM was performed during THA for patients with risk factors for iatrogenic nerve injury, and no permanent postoperative neuropathy occurred, despite the higher incidence reported in cases with risk factors. Intraoperative nerve monitoring can be an effective tool for preventing peripheral nerve injury during THA.

Peripheral nerve injury during THA can occur due to direct trauma. In patients with DDH, the sciatic nerve is closer to the ilium and ischium [24]. Additionally, malposition and adhesion of the nerves around the hip are common in patients with a history of hip surgery, including revision cases [10]. These factors increase the risk of intraoperative nerve injury caused by direct trauma, such as from a retractor. Therefore, nerve monitoring should be performed during THA in patients with DDH or a history of hip surgery. However, peripheral nerve injury during THA can occur in various circumstances, and it may be difficult to immediately detect nerve damage with fixed-point monitoring using MEP. We experienced a case with postoperative neurological symptoms, wherein the detection of intraoperative nerve damage was delayed because the MEP monitoring was performed only during surgical procedures with a high risk of intraoperative nerve injury, such as leg lengthening. Fortunately, the neuropathy was transient; however, the lag between the changes in nerve function and monitoring may result in a failure to prevent irreversible nerve injury. Therefore, after the abovementioned case, we adopted a monitoring method that tracks MEP at regular intervals throughout THA surgery. Furthermore, to compensate for the limitation of MEP being influenced by physiological factors such as hypotension and hypothermia [25], fEMG monitoring, which provides more continuous and physiological monitoring of nerve function [10], was combined with MEP. These monitoring techniques enabled the early detection of intraoperative nerve damage, allowing us to successfully avoid postoperative neuropathy. Thus, continuous IONM is recommended during THA for patients with risk factors for peripheral nerve injury caused by direct trauma.

With regard to leg lengthening, there is no definite consensus about the safety range of this procedure. Edwards et al. reported that the mean amount of leg lengthening that increased the risk of nerve injury was 2.7 and 4.4 cm for the peroneal and sciatic nerves, respectively [1]. Therefore, they concluded that leg lengthening should be limited to 4 cm. Johanson et al. reported that nerve palsy occurred in five of 34 patients who underwent THA due to leg lengthening of more than 2 cm [5]. Conversely, Eggli et al. reported no significant correlation between the amount of leg lengthening and nerve injury in DDH patients [26]. In this study, with the exception of those who received a subtrochanteric osteotomy, the mean amount of leg lengthening in primary THA for the patients without previous hip surgery was 1.9 cm (range 0.4-4.1 cm), and there were no intraoperative potential changes or postoperative nerve complications due to the leg lengthening regardless of the amount of lengthening that was undertaken. The MEP changes due to leg lengthening were observed in two of seven patients with a history of hip surgery. In such patients, nerve adhesion can occur due to previous surgeries, increasing vulnerability to nerve traction [6, 27]. Therefore, in THA for patients with a history of hip surgery, leg lengthening is a significant risk factor for intraoperative nerve injury, and IONM should be performed regardless of the amount of leg lengthening.

This study has several limitations. First, as a retrospective study with a relatively small cohort, the generalizability of the findings is limited. Additionally, the absence of a control group precludes definitive conclusions regarding the efficacy of IONM. Despite these limitations, however, this study suggests that IONM may contribute to preventing peripheral nerve injury during THA in patients with risk factors. Further studies with larger sample sizes, comparisons with a control group, and prospective designs are expected to strengthen the evidence for the effectiveness of IONM and contribute to the establishment of standardized protocols for its use in THA. Secondly, the MEP threshold was based on the criteria used in spinal surgery, but its applicability to THA remains unclear. Although no permanent postoperative neuropathy was observed in this study, we do not consider the threshold to be inappropriate. However, further research is necessary to validate its justification and to establish an appropriate MEP threshold specifically for THA. Thirdly, the intraoperative status of sensory nerves was not evaluated because both MEP and fEMG are monitoring methods to evaluate motor function. Consequently, we were unable to predict the occurrence of postoperative sensory disturbance, and one patient experienced transient postoperative numbness. Adding sensory nerve monitoring, such as SEP, to the regular-interval monitoring method mentioned in this study may facilitate more sensitive detection of the influence of the surgical procedure on the nerves.

## Conclusions

Intraoperative nerve monitoring is an effective tool for preventing peripheral nerve injury during THA in patients with risk factors. Since the risk of intraoperative nerve injury is higher due to nerve malposition and vulnerability to traction, IONM should be considered a routine technique, particularly in patients with DDH or a history of hip surgery. Given the various circumstances in which nerve injury can occur during THA, continuous IONM, including regular-interval MEP monitoring and fEMG, is essential for early detection of nerve damage and enables timely interventions to prevent irreversible postoperative neuropathy.
